# Abdominal Wall Erythema as a Suspected Extracardiac Manifestation of Cardiac Allograft Rejection

**DOI:** 10.7759/cureus.112218

**Published:** 2026-07-07

**Authors:** Yamann Sahlool, Fady Khoury, Andre Akhondi

**Affiliations:** 1 Cardiology, Los Robles Regional Medical Center, Thousand Oaks, USA; 2 Internal Medicine, Los Robles Regional Medical Center, Thousand Oaks, USA; 3 Cardiology, University of California, Los Angeles Medical Center, Los Angeles, USA

**Keywords:** antibody-mediated rejection, cardiac allograft, cellulitis mimic, extracardiac manifestation, heart transplantation, tacrolimus, transplant rejection

## Abstract

In a heart transplant recipient, a new cutaneous finding can be difficult to attribute to a single cause, and the clinical context does not always point to localized skin disease. We describe the case of a 54-year-old man five years after orthotopic heart transplantation who presented with severe periumbilical pain, hypoxemia, and abdominal wall erythema one week after completing intravenous immunoglobulin and rituximab for biopsy-proven antibody-mediated rejection (AMR). Computed tomography suggested paraumbilical cellulitis and possible pulmonary infection, and empiric antibiotics were appropriately initiated. However, the erythema was non-purulent and non-necrotizing, blood cultures were negative, there was no clear bacterial skin source, the patient’s atypical pretransplant ischemic pain phenotype had recurred, and the tacrolimus trough was subtherapeutic at 3.4 ng/mL, together raising concern for ongoing allograft immune injury. This case supports treating apparent cellulitis while urgently considering an extracardiac manifestation of graft rejection when support for a localized infectious source is weak and the transplant context is high risk. Preserved left ventricular ejection fraction does not exclude recurrent AMR or cardiac allograft vasculopathy, and early transplant-center coordination remains the critical management step.

## Introduction

Cutaneous findings in solid-organ transplant recipients are common and most often reflect infection, medication toxicity, malignancy, or an inflammatory dermatosis [1]. Far less frequently, skin findings in a heart transplant recipient may coincide with, or serve as a clue to, systemic alloimmune activity or to immune phenomena triggered by rejection-directed therapy. The literature describing true cutaneous correlates of isolated cardiac allograft rejection is sparse, which makes recognition of this possibility difficult at the bedside.

This difficulty is amplified when a cutaneous finding arises in a high-risk window. Antibody-mediated rejection (AMR) is a microvascular and endothelial alloimmune injury pattern whose diagnosis requires integration of clinical status, donor-specific antibody (DSA) testing, endomyocardial biopsy, histopathology, and immunopathology [2-4]. In brief, AMR is an alloimmune injury in which recipient antibodies bind the graft vascular endothelium, activating complement and producing microvascular injury that can compromise the graft even when contractile function appears preserved. DSA are recipient antibodies directed against donor human leukocyte antigens, and their presence supports an antibody-mediated process. Endomyocardial biopsy, the catheter-based sampling of right ventricular myocardium, provides the histologic and immunopathologic confirmation central to the diagnosis. Because the transplanted heart is surgically denervated, it does not transmit typical anginal pain, so ischemia or rejection often produces no chest pain and instead declares itself through vague or atypical symptoms or through objective testing. In a recently treated AMR patient with subtherapeutic immunosuppression, an apparent skin infection cannot safely close the diagnostic process. Both recurrent AMR and cardiac allograft vasculopathy (CAV) remain leading causes of late graft dysfunction and mortality after heart transplantation, and both can be clinically silent or present with nonspecific, atypical symptoms rather than overt heart failure [5,6]. We present a case in which abdominal wall erythema functioned as a possible extracardiac warning sign of ongoing graft immune vulnerability.

## Case presentation

**Figure 1:**
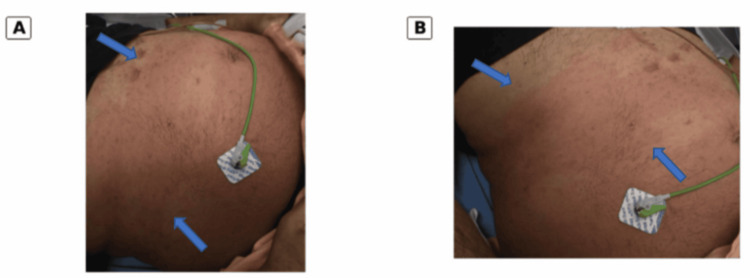
Abdominal wall erythema. (A) Clinical photograph demonstrating broad erythema involving the right and central abdominal wall. (B) Alternate view showing erythema across the right lateral and central abdomen. Blue arrows indicate the erythematous abdominal wall skin. No purulence, vesiculation, fluctuance, skin breakdown, or necrosis is visible.

A 54-year-old man presented with abrupt, severe periumbilical pain radiating to the back, dyspnea, and hypoxemia requiring up to 4 L/minute of oxygen by a nasal cannula. On arrival, he was afebrile at 36.8°C, with a heart rate of 88 beats per minute, blood pressure of 132/78 mmHg, a respiratory rate of 20 breaths per minute, and an oxygen saturation of 91% on room air that improved to 96% on 4 L/minute by a nasal cannula. He denied classic substernal pressure, orthopnea, cough, rhinorrhea, pleuritic pain, dysuria, sick contacts, and recent antibiotic exposure. He was afebrile before arrival and remained afebrile during early evaluation. Examination demonstrated broad, poorly circumscribed erythema and warmth involving the periumbilical and right abdominal wall, without crepitus, fluctuance, purulence, vesiculation, skin breakdown, or necrosis (Figure 1). The abdomen was soft and without peritoneal signs. A key historical detail was that his original pretransplant left anterior descending (LAD) artery infarction had also presented predominantly as abdominal and periumbilical pain rather than as typical angina.

Five years earlier, he had undergone orthotopic heart transplantation (OHT) at a tertiary medical center after acute LAD occlusion complicated by cardiogenic shock requiring prolonged Impella support. Maintenance immunosuppression included tacrolimus, sirolimus, and prednisone. Approximately one month before this admission, he had been diagnosed with biopsy-proven AMR and was treated with a course of intravenous immunoglobulin (IVIG) and rituximab. This rejection-directed therapy was completed approximately seven days before the current presentation, so that the abdominal wall erythema and periumbilical pain developed roughly one week after the final dose of AMR-directed treatment. Additional history included hypertension, obesity, and dyslipidemia treated with aspirin, rosuvastatin, and evolocumab.

The differential diagnosis included abdominal wall cellulitis adjacent to a small paraumbilical hernia, pneumonia or aspiration in an immunosuppressed host, gastroenteritis or duodenitis, biliary disease, renal parenchymal injury or pyelonephritis, CAV or acute coronary syndrome, and ongoing or recurrent allograft rejection with a suspected extracardiac or cutaneous manifestation.

Initial laboratory testing showed a white blood cell count of 8.8 x 10³/µL, creatinine 1.61 mg/dL, lactate 1.7 mmol/L, B-type natriuretic peptide 29 pg/mL, and high-sensitivity troponin 45 to 46 ng/L without a dynamic rise. C-reactive protein was less than 2.9 mg/L, and erythrocyte sedimentation rate was 23 mm/hour. Blood cultures remained negative. Severe acute respiratory syndrome coronavirus 2 and influenza testing were negative, and urinalysis was not supportive of urinary infection. Pneumococcal and *Legionella* urinary antigen testing were negative. The tacrolimus trough was subtherapeutic at 3.4 ng/mL. Laboratory values, with units and reference ranges, are summarized in Table 1. The largely normal infectious and inflammatory studies argued against an acute bacterial or viral source for the presentation.

**Table 1:** Laboratory values on admission with reference ranges and units.

Test	Result	Reference range	Units
White blood cell count	8.8	4.0–11.0	×10³/µL
Creatinine	1.61	0.7–1.3	mg/dL
Lactate	1.7	0.5–2.2	mmol/L
B-type natriuretic peptide	29	<100	pg/mL
High-sensitivity troponin	45–46 (no dynamic change)	0–78	ng/L
C-reactive protein	<2.9	<10	mg/L
Erythrocyte sedimentation rate	23	0–20	mm/hour
Tacrolimus trough	3.4 (subtherapeutic)	5–20	ng/mL
Blood cultures	Negative	Negative	—
SARS-CoV-2 polymerase chain reaction	Negative	Negative	—
Influenza polymerase chain reaction	Negative	Negative	—
Pneumococcal urinary antigen	Negative	Negative	—
*Legionella* urinary antigen	Negative	Negative	—
Urinalysis	No evidence of infection	Negative	—

**Figure 2:**
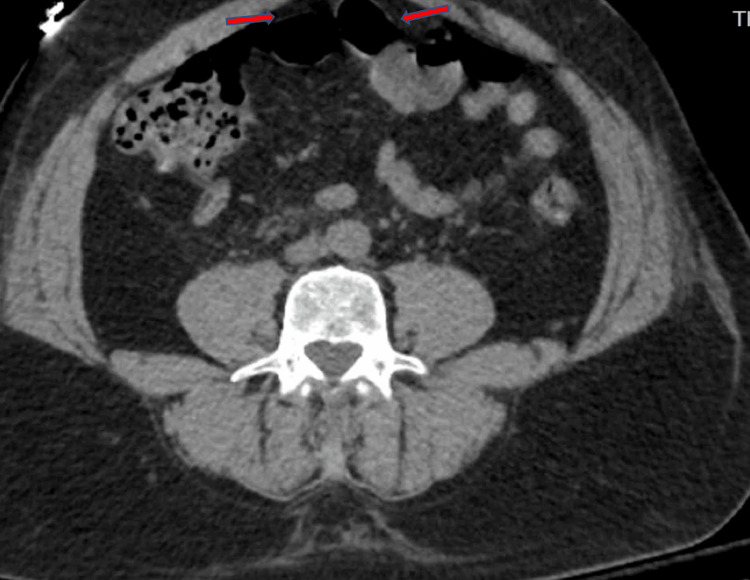
Computed tomography of the abdomen and pelvis. Axial image at the umbilical level showing a tiny fat-containing paraumbilical hernia with adjacent skin thickening and inflammation (arrows).

**Figure 3:**
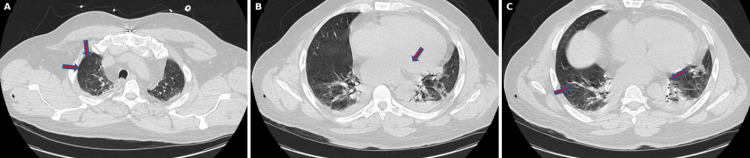
Computed tomography of the chest (lung windows). (A) Faint right-upper-lobe ground-glass and mosaic attenuation. (B) Lingular consolidation silhouetting the left heart border. (C) Dense, dependent bibasilar consolidation. Arrows indicate the findings.

**Figure 4:**
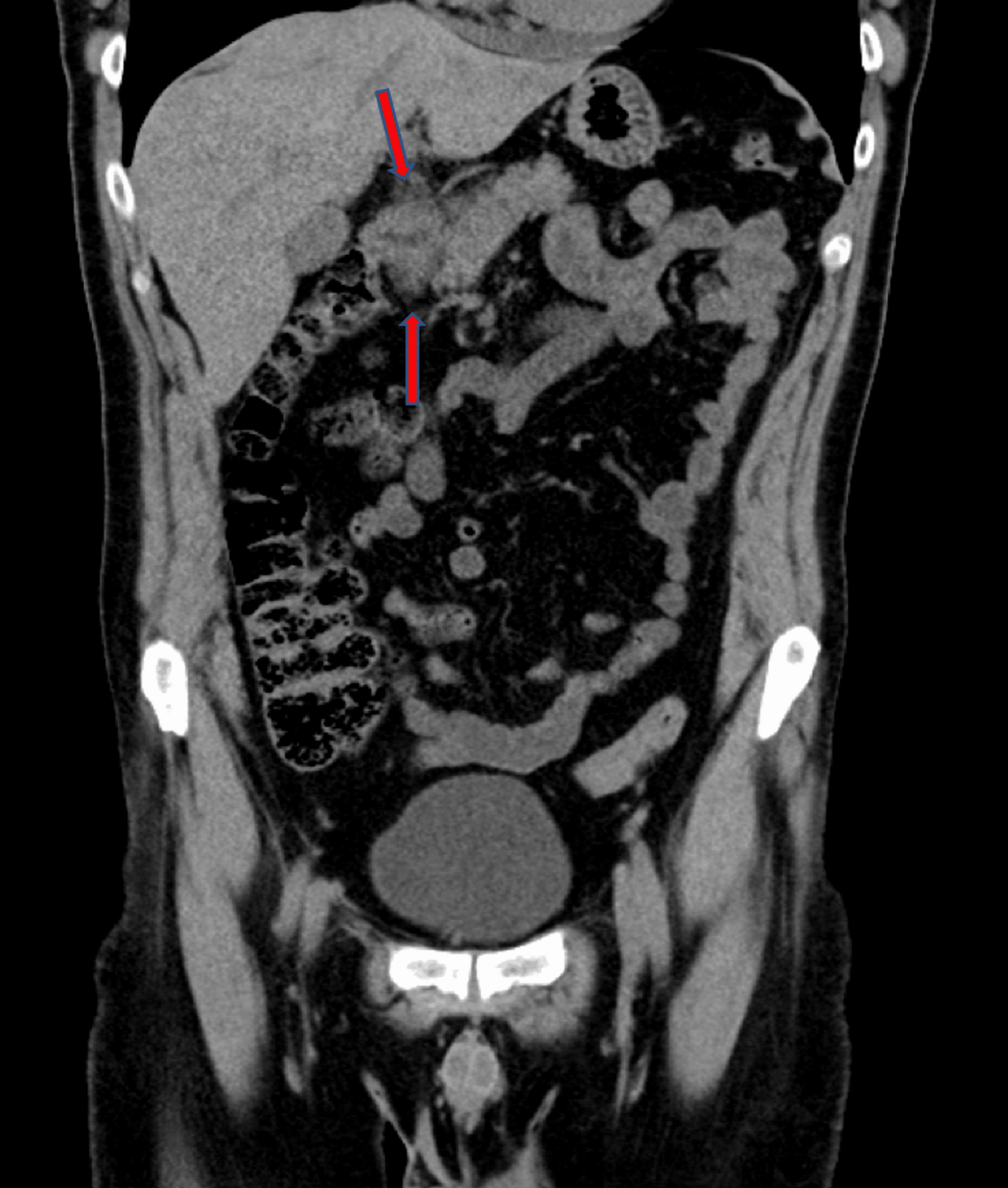
Magnetic resonance imaging of the abdomen. Coronal image demonstrating duodenal wall edema and thickening consistent with duodenitis (arrows).

Computed tomography (CT) of the abdomen and pelvis showed a tiny fat-containing paraumbilical hernia with adjacent skin thickening and inflammation (Figure 2), interpreted as potentially reflecting cellulitis, along with scattered indeterminate hepatic lesions. CT of the chest demonstrated faint right-upper-lobe ground-glass and mosaic attenuation and dense lingular and bibasilar consolidation (Figure 3), favored to represent atelectasis with superimposed infection not excluded. Magnetic resonance imaging of the abdomen showed possible duodenitis (Figure 4) and bibasilar airspace opacities greater than expected for atelectasis. Esophagogastroduodenoscopy demonstrated non-severe reflux esophagitis and gastritis without gross duodenal lesions. A hepatobiliary iminodiacetic acid scan was normal.

**Figure 5:**
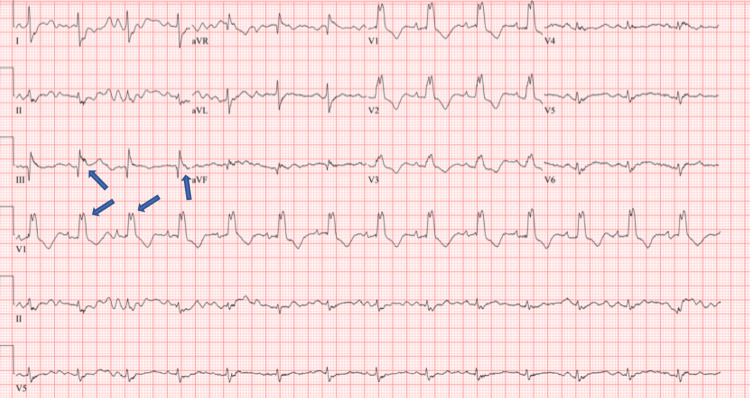
Twelve-lead electrocardiogram. Sinus rhythm with right bundle branch block, a QRS duration of approximately 144 ms, discordant repolarization abnormalities, and inferior Q waves. Blue arrows highlight the rsR′ (right bundle branch block) morphology in lead V1 and the inferior Q waves in leads III and aVF.

**Figure 6:**
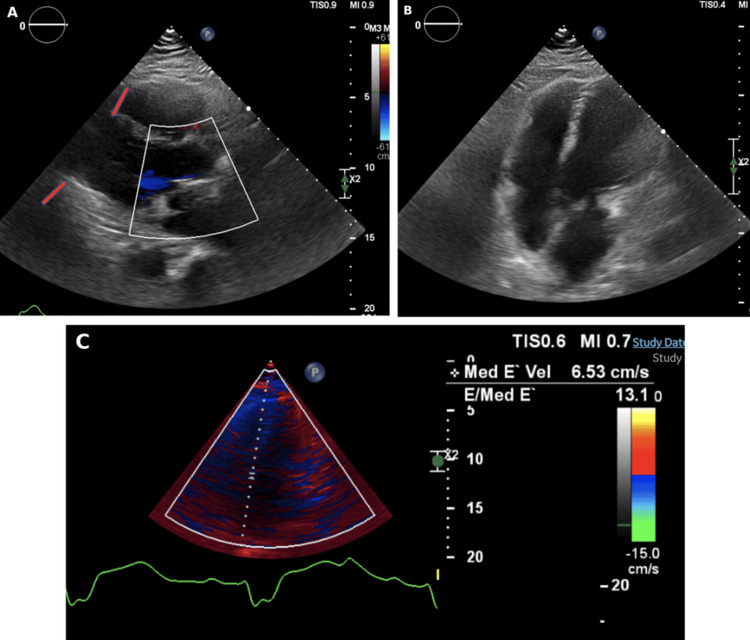
Transthoracic echocardiography. (A) Parasternal long-axis view; arrows indicate the moderately increased left ventricular wall thickness. (B) Apical four-chamber view showing normal left ventricular chamber size and moderate left atrial dilation. (C) Tissue Doppler imaging with a medial (septal) E/e′ of 13.1, consistent with elevated left ventricular filling pressure.

The electrocardiogram demonstrated sinus rhythm with right bundle branch block, a QRS duration of approximately 144 ms, discordant repolarization abnormalities, and inferior Q waves (Figure 5). Transthoracic echocardiography showed normal left ventricular (LV) chamber size, moderately increased LV wall thickness, an LV ejection fraction (LVEF) of 65% to 70%, no focal regional wall-motion abnormality, moderate left atrial dilation, trace tricuspid regurgitation, a right ventricular systolic pressure of 30 mmHg, and no pericardial effusion (Figure 6). The septal E/e′ was 13.1, and the mean E/e′ was 10.75.

Empiric antimicrobial therapy was initiated because the patient was immunosuppressed, CT suggested abdominal wall cellulitis, and chest imaging could not exclude pneumonia or aspiration. He received broad-spectrum antibiotics, the abdominal wall borders were monitored, and infectious diseases was consulted. Pneumonia serologies and urine antigens were sent. Gastroenterology evaluated the abdominal pain.

From a cardiology and transplant standpoint, the presentation could not be treated as isolated cellulitis. The pain reproduced the patient’s atypical ischemic phenotype, he had completed treatment for biopsy-proven AMR only one week earlier, and tacrolimus exposure was subtherapeutic. Immunosuppression with tacrolimus, sirolimus, and prednisone was continued without interruption. Echocardiography showing preserved LVEF and no wall motion abnormality made emergent angiography less compelling locally, but this did not exclude recurrent AMR, early graft dysfunction, or CAV. Cardiology recommended urgent transfer to the originating transplant center for graft-directed evaluation, including DSA assessment, endomyocardial biopsy, coronary evaluation as indicated, and transplant-directed therapy. While awaiting transfer, the team maintained telemetry, strict intake and output monitoring, electrolyte repletion, and avoidance of hypotension to preserve graft perfusion.

The patient had relative improvement in abdominal pain, was weaned to room air, and blood cultures remained negative. Because the transfer was delayed and a transplant follow-up was already scheduled within the week, he was discharged on oral amoxicillin-clavulanate and doxycycline with explicit instructions to follow up with the transplant team and to return immediately for worsening symptoms.

## Discussion

This case should be framed as a suspected extracardiac manifestation of an active allograft immune state rather than as routine cellulitis. The abdominal erythema was clinically important because it appeared in a narrow, high-risk window: one week after IVIG and rituximab for biopsy-proven AMR, with a tacrolimus trough of 3.4 ng/mL and recurrence of the patient’s prior atypical ischemic pain pattern. The literature on true cutaneous manifestations of isolated cardiac allograft rejection is sparse, which is precisely why the case is worth reporting. To our knowledge, this is among the first reports to frame abdominal wall erythema as a possible extracardiac warning sign of active cardiac allograft immune injury, distinct from the better-recognized cutaneous complications of transplantation and from eruptions caused by rejection-directed agents, and it offers a transferable bedside lesson that preserved ejection fraction does not exclude recurrent AMR or CAV. A prior heart-transplant case described biopsy-proven acute cellular rejection occurring with worsening atopic dermatitis despite preserved biventricular function and unremarkable blood testing, supporting the concept that clinically apparent skin inflammation and graft rejection can coexist in ways not captured by routine cardiac markers [7].

The differential remains rigorous. Solid-organ transplant recipients have broad dermatologic complications, including infection, malignancy, medication toxicity, and inflammatory dermatoses [1]. In addition, cutaneous eruptions may complicate rejection-directed therapy itself; serum sickness after thymoglobulin for cardiac allograft rejection has been reported with blanching erythematous macular eruptions that may begin in intertriginous and periumbilical regions [8]. This patient had not received thymoglobulin, but such reports establish that, in heart-transplant patients, abdominal or truncal erythema after rejection therapy cannot be dismissed as simple cellulitis without asking whether it reflects a broader immune process.

The strongest transplant-cardiology argument is mechanistic and temporal. AMR is fundamentally a microvascular and endothelial alloimmune injury pattern requiring integration of clinical status, DSA testing, endomyocardial biopsy, histopathology, and immunopathology [2-4]. Accordingly, the skin finding did not prove AMR, and the modestly elevated inflammatory markers did not localize inflammation to the abdominal wall. Instead, the erythema functioned as an extracardiac warning sign in a patient already at high risk for recurrent graft injury. Preserved LVEF was reassuring but not definitive. AMR is fundamentally an injury of the microvasculature and endothelium, so antibody-mediated graft injury can be active at the capillary level while global systolic function, which LVEF reflects, remains normal. Diastolic dysfunction and elevated filling pressures often precede any fall in ejection fraction, and in this patient, the septal E/e′ of 13.1 suggests an early diastolic abnormality despite an LVEF of 65% to 70% [9]. Similarly, CAV is a diffuse, concentric intimal disease of both the epicardial and microvascular coronary bed that produces chronic ischemia. Because the transplanted heart is denervated, it is frequently clinically silent and commonly presents with preserved LVEF and vague or atypical symptoms, such as abdominal pain, fatigue, or arrhythmia, or with sudden death rather than classic angina, and routine transthoracic echocardiography lacks sensitivity for early disease [5,6]. For these reasons, a normal LVEF and the absence of regional wall-motion abnormality cannot exclude recurrent AMR or CAV. Definitive exclusion requires endomyocardial biopsy, DSA testing, and dedicated coronary evaluation [10]. Because endomyocardial biopsy is invasive, carries procedural risk, and samples the myocardium focally, there is growing interest in noninvasive biomarkers of graft injury. Donor-derived cell-free DNA (dd-cfDNA) is one such marker: injured graft cells release donor-origin DNA fragments into the recipient circulation, and a rising dd-cfDNA fraction can signal ongoing allograft injury before a decline in ejection fraction or overt decompensation [11]. This is directly relevant to our patient, in whom LVEF was preserved, yet the temporal and mechanistic context raised concern for active immune injury, illustrating the surveillance gap that noninvasive markers are intended to close.

This report has limitations. It describes a single patient with a suspected, rather than histologically proven, extracardiac manifestation of allograft rejection, and a definitive causal link between the abdominal wall erythema and graft rejection cannot be established. Alternative or coexisting explanations remain possible, including bacterial cellulitis, a drug reaction or drug hypersensitivity, a serum sickness-like reaction to recent rejection-directed therapy, and a primary inflammatory dermatosis. Confirmatory graft-directed and dermatologic-immunologic testing that might have adjudicated among these possibilities was completed at the transplant center and was not available to the reporting team.

The management lesson is direct. In an OHT recipient with recent AMR, subtherapeutic tacrolimus, atypical abdominal pain, and abdominal wall erythema with weak objective support for a localized bacterial source, antibiotics are necessary but insufficient. The correct endpoint is not bedside certainty that the rash represents rejection; it is urgent coordination with the transplant center for biopsy, antibody assessment, and coronary evaluation so that recurrent AMR or CAV is not missed. Definitive graft-directed testing was completed at the originating transplant center after this admission. After transfer and discharge from the tertiary transplant center, the patient was contacted at follow-up. He reported feeling better, his immunosuppression had been increased, and the abdominal wall erythema improved following these medication changes. Definitive graft-directed testing, including DSA assessment and endomyocardial biopsy, was completed at the originating transplant center after this admission. The detailed results of that evaluation were managed by the transplant center and were not available to the reporting team.

## Conclusions

Abdominal wall erythema in a heart-transplant recipient recently treated for AMR should be treated as an infection when clinically suspected, but it should also prompt consideration of an extracardiac manifestation of ongoing allograft immune injury when the bacterial source is weak and immunosuppressive exposure is subtherapeutic. Apparent cellulitis in this setting should not close the diagnostic process when recent AMR, subtherapeutic tacrolimus, atypical ischemic symptoms, and weak support for a localized bacterial source coexist. Preserved LVEF does not exclude recurrent AMR or CAV, and early transplant-center coordination remains the critical management step.
